# A Rare Cause of Gastrointestinal Bleeding: Taeniasis in a Roux-en-Y Gastric Bypass Patient

**DOI:** 10.7759/cureus.108340

**Published:** 2026-05-06

**Authors:** Rita Ribeiro Dias, Paula Martins, André Pereira, Ana Fareleira, Silvestre Carneiro

**Affiliations:** 1 General Surgery, São João Local Health Unit, Porto, PRT; 2 Surgery, University of Porto, Porto, PRT

**Keywords:** anemia, gastrointestinal bleeding, roux-en-y gastric bypass, taenia infection, taeniasis

## Abstract

*Taenia *infection is a globally distributed parasitic disease that is uncommon in developed countries. Taeniasis typically presents with mild or no symptoms, and anemia is not a usual feature.

We report the case of a 50-year-old woman with a history of Roux-en-Y gastric bypass who presented with a history of abdominal pain, asthenia, and melena. Blood tests revealed severe anemia, prompting endoscopic evaluation. EGD showed only postsurgical changes, and lower endoscopy revealed no bleeding source. Video capsule endoscopy demonstrated blood near the Roux-en-Y anastomosis and in the ileum. Enteroscopy subsequently identified a duodenal ulcer with recent bleeding and a tapeworm in the afferent limb. Hemostasis was achieved with hemostatic powder, and the patient was treated with praziquantel. At follow-up, she was asymptomatic, and her hemoglobin levels had normalized.

Taeniasis is a rare cause of gastrointestinal bleeding, with only a few cases described in the literature. In this patient, the parasite was detected in the excluded limb of the gastric bypass. Mucosal injury caused by parasite attachment or movement may explain the bleeding. This case highlights the importance of considering parasitic infections in the differential diagnosis of melena, even in patients from non-endemic regions and without typical risk factors.

Increased awareness of this rare presentation is essential to ensure timely diagnosis and treatment.

## Introduction

*Taenia *infection is a parasitic zoonosis with worldwide distribution, most common in developing countries [1,2].

Human infection can occur through the ingestion of eggs from the adult worm, leading to cysticercosis, or through the ingestion of cysticerci in raw or undercooked meat, which results in infestation by the adult tapeworm in the gastrointestinal tract, a condition known as taeniasis [3,4].

Taeniasis is mostly asymptomatic, but it may cause abdominal pain, nausea, anorexia, and weight loss [2,5,6]. Some patients also report the presence of proglottids in their feces [1,7]. Although complications are uncommon, gastrointestinal bleeding is a rare but possible manifestation.

We report a case of an unusual presentation of *Taenia *infection associated with gastrointestinal bleeding in a patient with a Roux-en-Y gastric bypass, with particular interest given the visualization of the parasite in the afferent limb.

## Case presentation

We present the case of a 50-year-old woman with a medical history of Roux-en-Y gastric bypass performed one year earlier. The patient reported progressive asthenia and diffuse abdominal pain over approximately three weeks, followed by the onset of melena three days prior to admission, which prompted evaluation in the emergency department. She denied the use of nonsteroidal anti-inflammatory drugs, anticoagulants, or alcohol consumption. There was no history of recent travel or ingestion of raw meat. 

The patient was hemodynamically stable. Abdominal examination revealed a soft, non-tender abdomen without pain on palpation. Digital rectal examination (DRE) was positive for melena.

Blood tests showed anemia, with a hemoglobin level of 7.1 g/dL. Previous results from two weeks earlier showed a hemoglobin level of 11 g/dL.

The patient underwent a blood transfusion and esophagogastroduodenoscopy (EGD), which revealed postoperative changes consistent with the known Roux-en-Y gastric bypass. The gastrojejunal anastomosis was widely patent and free of ulceration, and the efferent limb was also patent without abnormalities. Lower gastrointestinal endoscopic examination revealed no lesions, showing only digested blood in the terminal ileum.

**Figure 1:**
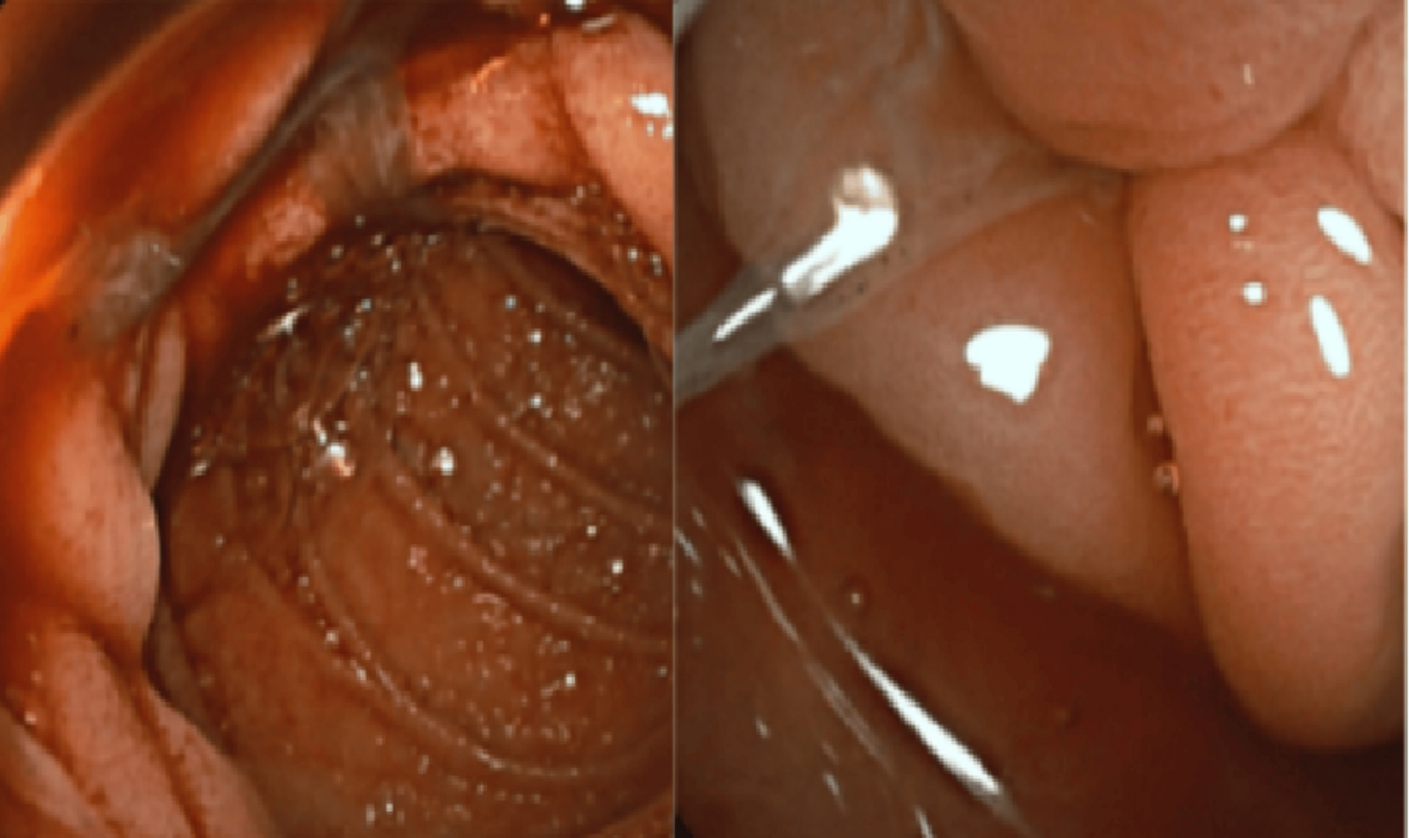
Images from enteroscopy showing the presence of tapeworm in the afferent limb

After the initial upper and lower endoscopic evaluations were inconclusive, video capsule endoscopy was performed to investigate a possible small bowel source of bleeding. This demonstrated blood near the Roux-en-Y anastomosis and within the ileum. Given the ongoing evidence of bleeding and the capsule endoscopy findings, enteroscopy was pursued to allow both diagnostic evaluation and potential therapeutic intervention. During retrograde examination of the duodenum through the afferent limb of the Roux-en-Y anastomosis, a duodenal ulcer (Forrest IIb) was identified along with a tapeworm, as shown in Figure 1, which is of particular interest given the visualization of the parasite in the afferent limb. The ulcer showed signs of recent hemorrhage and was treated with hemostatic powder.

The patient was evaluated by an infectious diseases specialist and treated with praziquantel. She remained clinically stable and asymptomatic during 48 hours of inpatient monitoring and was subsequently discharged.

She was seen at a follow-up visit 10 months later and was asymptomatic, with no further episodes of melena or anemia. She underwent a new EGD, which was unremarkable.

## Discussion

Given the presence of melena and anemia in a patient with prior Roux-en-Y gastric bypass, the main differential diagnoses initially considered were marginal ulceration, bleeding from the excluded stomach or duodenum, and small bowel angiodysplasia. Evaluation of these patients is particularly challenging because the altered gastrointestinal anatomy limits access to the excluded limb by conventional endoscopy.

Despite initially negative upper and lower endoscopic evaluations, further investigation with capsule endoscopy and subsequent enteroscopy was performed due to ongoing evidence of bleeding. Retrograde enteroscopy through the afferent limb ultimately identified a duodenal ulcer with signs of recent hemorrhage associated with a tapeworm. To our knowledge, this is the first reported case describing gastrointestinal hemorrhage associated with *Taenia *infection identified within the excluded limb of a Roux-en-Y gastric bypass, highlighting the diagnostic complexity of gastrointestinal bleeding in this setting.

Anemia and overt bleeding are not typical manifestations of *Taenia *infection, which is usually asymptomatic or associated with nonspecific gastrointestinal symptoms [8]. Proposed mechanisms of bleeding include direct mucosal injury from scolex attachment, mechanical irritation due to parasite motility, and local inflammatory or immune-mediated responses leading to mucosal erosion [3,9].

Parasitic infections of the gastrointestinal tract are uncommon in developed countries, and therefore often not considered in the differential diagnosis of anemia or gastrointestinal bleeding [8,9,10]. Although ingestion of raw or undercooked meat is a well-known risk factor for taeniasis [1], our patient denied such exposure.

Regarding treatment, the standard approach is praziquantel 5-10 mg/kg as a single dose, which was the regimen used in this patient. Tapeworms are generally highly responsive to anti-helminthic therapy [3,5]. However, in this case, the parasite was located in the excluded portion of the gastrointestinal tract, which escapes standard endoscopic surveillance. The effectiveness of the treatment was confirmed by the resolution of the patient’s symptoms, normalization of her hemoglobin levels, and follow-up EGD showing no residual lesions or parasites.

## Conclusions

The aim of this report is to illustrate the clinical relevance of this rare condition in developed countries. Although uncommon, taeniasis should be considered as a possible cause of melena. In this case, the parasite was located in the afferent limb of a Roux-en-Y gastric bypass, which posed a particular diagnostic challenge due to the altered anatomy.
